# Event-Based Robotic Grasping Detection With Neuromorphic Vision Sensor and Event-Grasping Dataset

**DOI:** 10.3389/fnbot.2020.00051

**Published:** 2020-10-08

**Authors:** Bin Li, Hu Cao, Zhongnan Qu, Yingbai Hu, Zhenke Wang, Zichen Liang

**Affiliations:** ^1^JingDong Group, Beijing, China; ^2^Robotics, Artificial Intelligence and Real-time Systems, Technische Universität München, München, Germany; ^3^Computer Engineering and Networks Lab, Eidgenössische Technische Hochschule (ETH) Zürich, Zürich, Switzerland; ^4^College of Automotive Engineering, Tongji University, Shanghai, China

**Keywords:** neuromorphic vision sensor, SMP filter, event-grasping dataset, grasping detection, deep learning

## Abstract

Robotic grasping plays an important role in the field of robotics. The current state-of-the-art robotic grasping detection systems are usually built on the conventional vision, such as the RGB-D camera. Compared to traditional frame-based computer vision, neuromorphic vision is a small and young community of research. Currently, there are limited event-based datasets due to the troublesome annotation of the asynchronous event stream. Annotating large scale vision datasets often takes lots of computation resources, especially when it comes to troublesome data for video-level annotation. In this work, we consider the problem of detecting robotic grasps in a moving camera view of a scene containing objects. To obtain more agile robotic perception, a neuromorphic vision sensor (*Dynamic and Active-pixel Vision Sensor, DAVIS*) attaching to the robot gripper is introduced to explore the potential usage in grasping detection. We construct a robotic grasping dataset named *Event-Grasping* dataset with 91 objects. A spatial-temporal mixed particle filter (SMP Filter) is proposed to track the LED-based grasp rectangles, which enables video-level annotation of a single grasp rectangle per object. As LEDs blink at high frequency, the *Event-Grasping* dataset is annotated at a high frequency of 1 kHz. Based on the *Event-Grasping* dataset, we develop a deep neural network for grasping detection that considers the angle learning problem as classification instead of regression. The method performs high detection accuracy on our *Event-Grasping* dataset with 93% precision at an object-wise level split. This work provides a large-scale and well-annotated dataset and promotes the neuromorphic vision applications in agile robot.

## 1. Introduction

Neuromorphic vision based on neuromorphic sensors represents the visual information in the way of address-event-representation (AER). There is a growing interest coming from the computer science community, especially from the neuroscience and computer vision. In traditional computer vision, there are lots of widely used datasets, such as ImageNet (Deng et al., 2009), COCO (Lin et al., 2014), and KITTI (Geiger et al., 2013). These high-quality datasets serve as a standard platform for development and comparison of the state-of-the-art algorithms and methods in the computer vision field. However, neuromorphic vision datasets, especially for those with high quality, are still difficult to acquire. This indirectly reveals the fact that neuromorphic vision has not been widely studied. Robotic grasping plays a major role in complex tasks, such as human robot interaction (Bicchi and Kumar, 2000) and robotic assembly (Cutkosky, 2012). The Grasping dataset of Cornell's researchers was recorded with an RGB-D camera, which is widely used by researchers, and greatly contributes to the development in robotic grasping (Jiang et al., 2011). The grasping dataset demonstrates several good grasping positions and bad grasping positions for each view of an object as rectangular bounding boxes, which contributes greatly to training a parallel plate gripper (PPG) to perceptively grasp an object. The Yale human grasping dataset records human grasping behavior in unstructured environments with a head camera that is utilized to train dynamic manipulative behaviors spanning much of the typical human hand usage (Bullock et al., 2014). In Lenz et al. (2015), the researchers have proceeded with the idea of Jiang et al. (2011) and developed a two-step neural network. In Redmon and Angelova (2015), the authors combined the convolutional neural network (CNN) to treat the grasping information as neural output, and they accomplished some achievements. A layer-wise fusion method was proposed in Asif et al. (2018) to improve the performance of robotic grasping detection using state-of-the-art deep learning algorithms. In Wu et al. (2019), the authors also used a fusion approach to carry out a grasp-detection task. Specially, a hierarchical feature fusion is utilized to learn object detection and grasping pose estimation simultaneously. Moreover, a new pixel-wise grasp convolutional neural network was developed in Morrison et al. (2020) where grasp rectangles are predicted by directly regress a grasp map representation. Following the road map of Kumra et al. (2019), Wang et al. (2019), and Morrison et al. (2020) improved the performance of grasping detection with their generative convolutional neural network. However, the above research usually take RGB or RGB-D images as input to carry out grasp rectangle prediction. In this paper, we try to tackle robotic grasping problem using neuromorphic vision.

It is still far from a solved problem for robots to grasp with a high success rate, especially considering the resource constraints. It is hard to align a robot gripper with an ideal grasping position with visual sensors due to the lack of perception and the uncertainties of noisy measurements. Furthermore, it is difficult to get a balance between high-complexity perception algorithms and low computation, storage, and power consumption in an embedded robot system. These problems deteriorate especially when grasping a moving object. Meanwhile, the neuromorphic vision sensor is seldom applied in the field of robotics since it is difficult to annotate neuromorphic vision datasets with data format of asynchronous event stream. However, neuromorphic vision has its unique advantages for robotic applications if the dataset annotation problem could be appropriately solved. The researchers of Chen et al. (2018) applied neuromorphic vision sensor in intelligent transportation system. Some works have been reviewed in Chen et al. (2020) and Gallego et al. (2019). Considering the high requirement of calculation time and storage consumption, the traditional RGB-D camera cannot satisfy the real-time feature. Besides, a frame-driven camera can only capture a blur due to the large latency of processing dynamic frame (Barranco et al., 2014), which cannot adapt to the fast-moving object detection and tracking. In this paper, we build a faster sensing pipeline through a neuromorphic vision sensor: *Dynamic and Active-pixel Vision Sensor* (DAVIS). DAVIS only transmits the local pixel-level changes caused by lighting intensity changing in a scene *at the time they occur* in the way of a bio-inspired retina. Apart from the low latency, data storage and computational resources are drastically reduced due to the sparse event stream. Another key property is its very high dynamic range, which is 130 vs. 60 dB of frame-based vision sensors. These features have already made it useful in resource-limited applications where conventional frame-based cameras are typical not appropriate (Liu H. et al., 2016; Kueng et al., 2016). Compared with conventional frame-based camera (typically 30–100 ms), the DAVIS emitted events individually and asynchronously at the time they occur.

In this paper, we have created a robotic grasping dataset named “Event-Grasping Dataset” by directly shooting the real world with a neuromorphic vision sensor (DAVIS) and then making a label for the asynchronous event stream. Since demonstrating several good and bad grasping positions for each view of an object is challenging, especially in the asynchronous event stream, we designed an annotation system consisting of four LED lights. Besides, we further developed a particle filter algorithm to achieve fast and robust tracking of four LED light markers. With using the tracked LED trajectories, we annotated the good and bad grasping positions (four LEDs correspond to the four vertices of the grasping position). Lastly, we used a single-stage deep neural network for grasping detection. Our approach naturally captures grasp objects of various sizes with combining predictions from multiple feature maps with different resolutions. The deep network architecture achieves a better performance on our Event-Grasping Dataset by considering the angle learning problem to classification instead of regression.

The main content of this paper covers six parts. Section 2 presents the neuromorphic grasping system, including system settings and synchronization problem. In section 3, an event-based LED markers tracking method is introduced. Event-Grasping dataset is illustrated in section 4. Section 5 describes the details of our event-based grasping detection approach. Section 6 gives experiment results and an analysis on the Event-Grasping dataset. The conclusions of this work are discussed in section 7.

## 2. Neuromorphic Grasping System

In this section, the neuromorphic grasping system is introduced, and the principle of our Non-manual labeling method based on LED markers is presented.

### 2.1. System Setting

**Figure 1:**
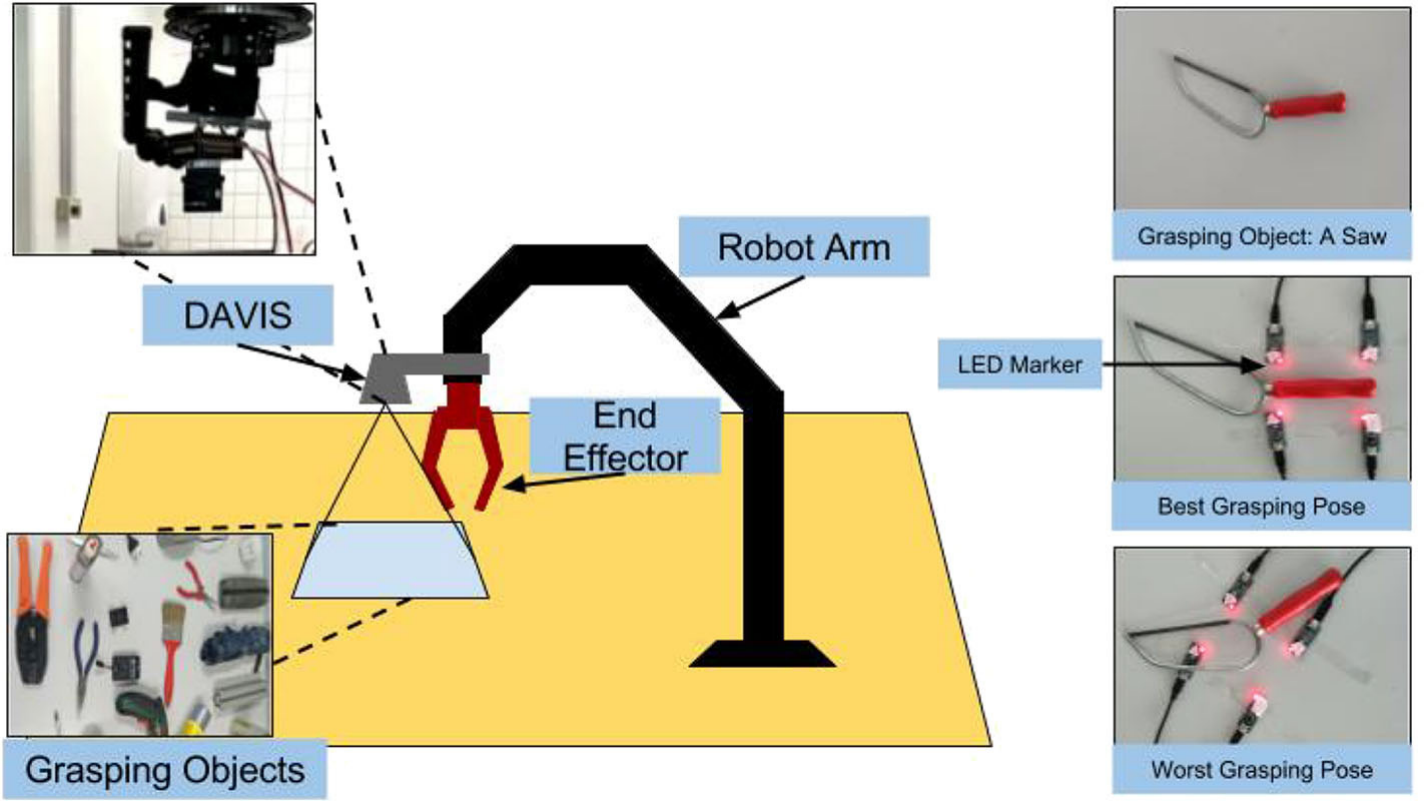
The hardware construction and the annotation setup demonstration.

In order to grasp the object, we need to obtain the direction vector between PPG and object so that the robot can approach the object. We consider the normal direction of the table surface as the direction vector, i.e., the gripper moving strictly vertically to the table. Under this situation, the rectangle placed on the table becomes a parallelogram in the view of the camera (DAVIS) when the camera lens plane is not parallel to the table surface. In our dataset, this parallelogram strategy will be set as the first annotation. However, this strictest situation will cause pixel extraction problem due to parallelogram. Therefore, another direction vector needs to be determined. To enlarge the diversity of our dataset, we will make a fine tuning of parallelogram to get a rectangular annotation dataset. Four LED markers are placed blinking at four different high frequencies near the object on a table to construct four vertexes of the grasping rectangle. By distinguishing different LEDs based on the time intervals between the ON and OFF events, it is able to track multiple markers without considering the data association problem. Through continuous tracking, the grasping rectangle can be automatically and continuously annotated in different view direction. The whole hardware and annotation setups are shown in Figure 1.

Compared with other grasping datasets, which are annotated manually with a rectangle in each static image, our neuromorphic grasping system has the ability to automatically annotate each grasping pose in a parallelogram dynamically at the time resolution of μs. The dataset construction can be divided into three steps: (1) record the annotation event data; (2) record the original object event data; and (3) extract grasping information and map it to the object data.

### 2.2. Synchronization Problem

The synchronization between the annotation event data and the object event data must be taken into consideration when we map the annotation to the object. Besides, the synchronization between blinking start and robot arm moving start must be also taken into account. A periodic moving approach is applied to solve the synchronization problem.

**Figure 2:**
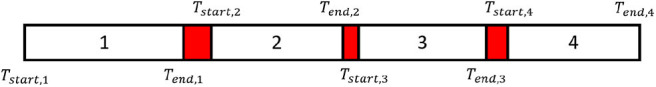
The four steps in the event data stream recording and their crucial time points. The red areas present the useless connection gaps between both successive steps. The start time of first step, *T*_*start*,1_, is also the start time of the entire recording stream; the end time of the fourth step, *T*_*end*,4_, is also the end time of the entire recording stream.

The manipulator maintains periodic rotating and tilting for four fixed steps when the camera records data, and each step lasts for two periods at least. In the first two steps, four LEDs with manually selected blinking frequencies are used to define a good grasping pose and a bad grasping pose for the object. In the last two steps, we remove the LEDs and only keep the object on the table to simulate the real grasping scenario. The third step records the dark lighting condition situation, and then the fourth step records the bright lighting condition. After recording, the start and end time points of each step are sought manually along the time axis in the jAER Trunk software (Llongi and Delbruck, 2017), see Figure 2.

If the period of robot arm motion is more precise than 1 ms, we can simply obtain the aligned event data stream: *t*_*i*_ ∈ [*T*_*end*,4_ − (*n*_*i*_ − 1) × *T, T*_*end*,4_ − *n*_*i*_ × *T*], where *t*_*i*_ is the time points of the *i*-th step; *T*_*end*,4_ is the end time point of the fourth step; *T* presents the period of robot arm's motion; *n*_*i*_ in the *i*-th step is the smallest integer that satisfies the following:

(1) $$\begin{array}{r} T_{end,4}-n_{i}\times T\leq T_{end,i} \end{array}$$

## 3. LED Marker Tracking

This section describes our event-to-window method for tracking LED positions from DAVIS event-based output. Our approach is collecting raw DAVIS event data sequence as input to the algorithm, processing a small part of the whole data sequence for each window cycle with sliding step of 1 *ms*, and eventually obtaining pixel-level positions of LED markers for each sliding window.

### 3.1. Event Data Formulation

Inspired by Censi et al. (2014), we propose a method to convert raw event data finally into interval data, which is more convenient to use for the rest LED tracking computation. This method incorporates four stages: Raw Events, States, Transitions, and Intervals, where States and Transitions are two intermediate types of data, and Intervals are actually hyper-transitions generated from transition data.

**Figure 3:**
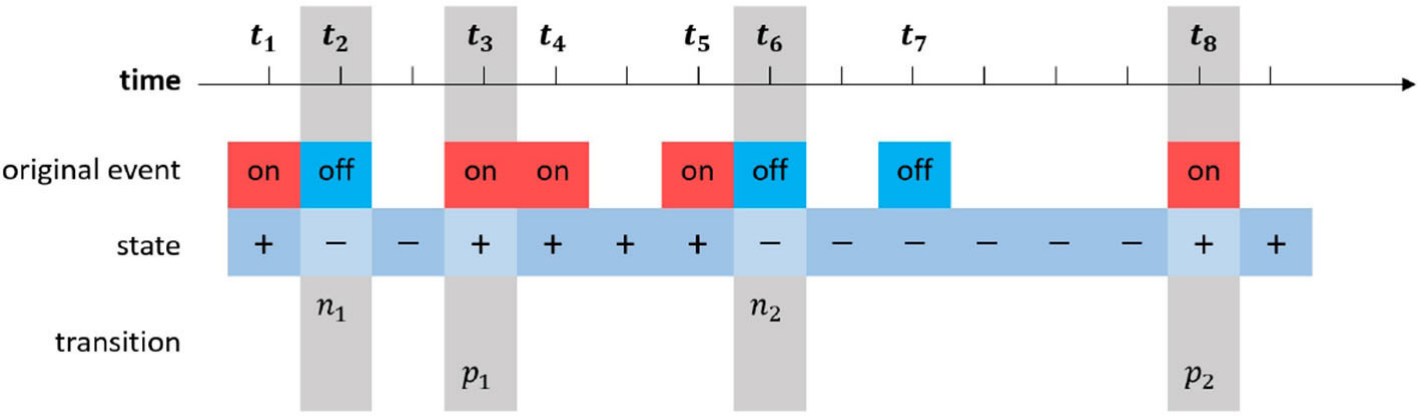
The raw event data stream, generated state data stream, and transition data stream of a single pixel. The transition data contain two types, “p” and “n.” Since only the transition interval between both successive transitions with the same type is taken into consideration (see Equation 2), the concrete polarity is not important to the tracking.

- **Raw Events**: The raw event data from DAVIS can be formed as a tuple: {*t*_*k*_, *l*_*k*_, (*x*_*k*_, *y*_*k*_)}. *k* is assumed to be the index of this event in the event stream. *t*_*k*_ represents the timestamp of the *k*-th event with unit of μs; *l*_*k*_ represents the polarity of the lighting intensity changing, *l*_*k*_ ∈ {“*on*,” “*off*”}; (*x*_*k*_, *y*_*k*_) represents the pixel coordinates (integer) in the scene, *x*_*k*_ ∈ [0...239] and *y*_*k*_ ∈ [0...179]. Since each event is triggered asynchronously, the array of *t*_*k*_ is not uniform distributed over time.
- **States**: The lighting intensity state represents the current brightness condition of each pixel when processing a single event. The state may vary only if there is a new event triggered in this pixel at the current timestamp. For each timestamp, each pixel has a lighting intensity state, which can be formed as a tuple: {*t*_*k*_, *s*_*i,k*_, (*x*_*i*_, *y*_*i*_)}. *t*_*k*_ represents the *k*-th timestamp in the event stream with unit of μs; *s*_*i,k*_ represents the lighting intensity state of the i-th pixel at timestamp *t*_*k*_, *s*_*i,k*_ ∈ {“+,” “−”}; (*x*_*i*_, *y*_*i*_) represents the coordinates, and the range of coordinates is similar as the tuple above.
- **Transitions**: Each time when the state changes, it will produce a transition. If the state is transformed from “−” to “+,” a positive transition is generated, and from “+” to “−” means negative transition. The transition data for i-th pixel at *t*_*k*_ can be formed as a tuple : {*t*_*k*_, *Tr*_*i,k*_, (*x*_*i*_, *y*_*i*_)}. *Tr*_*i,k*_ represents the transition at timestamp *t*_*k*_ in the *i*-th pixel, *Tr*_*i,k*_ ∈ {“*p*,” “*n*”}, where “p” for positive and “n” for negative. In practice, we merely need to store the last transition time of both “p” and “n” types for each pixel, which is the only important information when calculating transition intervals in the next stage. The figure of original event data, state data, and transition data is shown as Figure 3.
- **Intervals**: Transition intervals are calculated from two successive transitions of the same type and can be represented as a tuple: {*t*_*k*_, Δ_*Tr*_, (*x*_*i*_, *y*_*i*_)}, where Δ_*Tr*_ is the interval between transitions of the same type, and (*x*_*i*_, *y*_*i*_) are pixel coordinates. For example, if a new positive transition *p*_2_ is generated at *t*_8_, and the last positive transition *p*_1_ in this pixel happened at *t*_3_, the transition interval between both positive transitions can be calculated as
  (2) $$\begin{array}{r} \Delta_{Tr}=\Delta_{p}=t\left(p_{2}\right)-t\left(p_{1}\right)=t_{8}-t_{3} \end{array}$$

To get a better event streams, four blinking frequencies *T*_*l*_(*l* = 1, 2, 3, 4) are chosen as {3, 000, 3, 800, 4, 400, 5, 000}μs, which are applied to four LED markers, respectively. The blinking frequency is supposed to be higher than the moving frequency so that the two successive transitions with the same type can be ensured to take place at the same pixel.

### 3.2. Spatiotemporal Mixed Particle Filter

In this section, we will discuss the usage of Spatial-temporal Mixed Particle (SMP) filter inspired by Arulampalama et al. (2002), which has great advantages for tracking LED markers with low latency. DAVIS has a precise result with a sub-ms-cycle level, and the moving velocity of each marker is lower than 1 pixel/ms. We thus set the sliding window to be 10*ms* with sliding step of 1*ms*. In our experiment, we assigned 1,000 particles to the tracking of all four markers, represented as *j* = 0…1, 000, *l* = 0…4. Additionally, $x_{k,l}^{j}$ represents the coordinates of the particle (2-dimension vector) in the *k*-th cycle.

The weight for each particle consists of two parts of temporal evidence and spatial evidence; the particle weights are updated with the new likelihood and its last weight:

(3) $$\begin{array}{r} w_{k,l}^{j}=w_{k-1,l}^{j}\times \left(ET_{i,k,l}+\alpha \times ES_{i,k,l}\right) \end{array}$$

where α is the ratio between the evidence, and *ET*_*i,k,l*_ and *ES*_*i,k,l*_ are temporal evidence and spatial evidence, respectively, if the *i*-th pixel is occupied by the *l*-th marker during the *k*-th cycle. This strategy helps to increase the weights of desired pixels without any other direct affections on particle iteration or resampling. Based on the particle weights and the coordinates of particles in each set, the current position of each marker can be updated to realize multiple LED markers tracking. The whole algorithm of this particle filter is shown as Algorithm 1.

**Algorithm 1:** Spatiotemporal mixed particle filter.

**procedure** $\left({\left\{{\left\{x_{k,l}^{j},w_{k,l}^{j}\right\}}_{j=1}^{N_{s}}\right\}}_{l=1}^{4}\right)\;=\;$SMPF (${\left\{{\left\{x_{k-1,l}^{j},w_{k,l}^{j}\right\}}_{j=1}^{N_{s}}\right\}}_{l=1}^{4}$, {Δ_*Tr,k*_})
Calculate ${\left\{ET_{k.l}\right\}}_{l=1}^{4}$ based on {Δ_*Tr,k*_}
**for** *l* ← 1:4 **do**
**for** *i* ← (0, 0):(239, 179) **do**
**if** number of events in the i-th pixel < 3 **then**
*ET*_*i,k,l*_ ← 0
**end if**
**end for**
**end for**
**for** *l* ← 1:4 **do**
**for** *j* ← 1:*N*_*s*_ **do**
Draw $x_{k,l}^{j}\sim p\left(x_{k,l}|x_{k-1,l}^{j}\right)=N\left(x_{k-1,l}^{j},I\sigma^{2}\right)$
Get the particle's temporal evidence *ET*_*i,k,l*_ according to $x_{k,l}^{j}$
**end for**
**end for**
**for** *l* ← 1:4 **do**
*N*_*reselect*_ ← *Equation* (5)
**if** *N*_*reselect*_ < *Th*_*l*_ **then**
Run reselection (replacement)
Get the new particle set ${\left\{x_{k,l}^{j}\right\}}_{j=1}^{N_{s}}$ and the new temporal evidence set ${\left\{ET_{i,k,l}\right\}}_{i=1}^{N_{s}}$
**for** *j* ← 1:*N*_*s*_ **do**
$w_{k-1,l}^{j}\leftarrow 1/N_{s}$
**end for**
**end if**
Normalize ${\left\{ET_{i,k,l}\right\}}_{i=1}^{N_{s}}$
Calculate **μ*T*_*k,l*_** and **Σ*T*_*k,l*_** of the temporal evidence matrix *ET*_*k,l*_
**for** *j* ← 1:*N*_*s*_ **do**
Get spatial evidence *ES*_*i,k,l*_ according to $x_{k,l}^{j}$, $ES_{i,k,l}=N\left(x_{k,l}^{j}|\mu T_{k,l},\Sigma T_{k,l}\right)$
**end for**
Normalize ${\left\{ES_{i,k,l}\right\}}_{i=1}^{N_{s}}$
**for** *j* ← 1:*N*_*s*_ **do**
Update the particle's weight $w_{k,l}^{j}=w_{k-1,l}^{j}\times \left(ET_{i,k,l}+\alpha \times ES_{i,k,l}\right)$
**end for**
Normalize ${\left\{w_{k,l}^{j}\right\}}_{j=1}^{N_{s}}$
*N*_*eff*_ ← *Equation* (7)
**if** *N*_*eff*_ < *Th*_*eff*_ × *N*_*s*_ **then**
Run resampling
Get the new particle set ${\left\{x_{k,l}^{j}\right\}}_{j=1}^{N_{s}}$
**for** *j* ← 1:*N*_*s*_ **do**
$w_{k,l}^{j}\leftarrow 1/N_{s}$
**end for**
**end if**
Update the tracked position of the l-th LED marker, $x_{LED,k,l}=\overset{N_{s}}{\underset{j=1}{\sum }}w_{k,l}^{j}\times x_{k,l}^{j}$
**for** *j* ← 1:*N*_*s*_ **do**
$w_{k-1,l}^{j}\leftarrow w_{k,l}^{j}$
**end for**
**end for**
**end procedure**

#### 3.2.1. Temporal Evidence

Each transition interval contributes to all four temporal evidence matrices, which increases the robustness of the Gaussian noise shown in Censi et al. (2014). The temporal evidence Δ_*i,k,n*_ is expressed as

(4) $$\begin{array}{r} ET_{i,k,l}=\overset{N_{i,k}}{\underset{n=1}{\sum }}p\left(\Delta_{i,k,n}|T_{l}\right)=\overset{N_{i,k}}{\underset{n=1}{\sum }}N\left(\Delta_{i,k,n}|\mu_{l},\sigma_{l}^{2}\right) \end{array}$$

where Δ_*i,k,n*_ represents the *n*-th transition interval in the *i*-th pixel of the *k*-th cycle, and *i* ∈ (0, 0) ~ (239, 179) represents the coordinates of the pixel. *N*_*i,k*_ represents the total number of transition intervals in the *i*-th pixel taken place in the *k*-th window cycle. *T*_*l*_ is the *l*-th marker, and μ_*l*_ and $\sigma_{l}^{2}$ are the mean and the variance of its blinking period, respectively. Δ_*i,k,n*_ should obey this Gaussian probability distribution *N* when it results from this marker, and the sum of these probabilities results in the evidence *ET*_*i,k,l*_. The distribution of blinking transition intervals is a narrow Gaussian distribution which reduces the computation. The particle weights for each marker should be normalized to ensure the sum equals 1 before reselection.

Many particles have a zero temporal evidence, which means this particle set cannot cover enough valid measurements in the current cycle, and the current markers' pixels cannot therefore be detected through these particles. Whether a reselection process is required could thus be expressed as

(5) $$\begin{array}{r} N_{reselect}=\overset{N_{s}}{\underset{i=1}{\sum }}ET_{i,k,l}<Th_{l} \end{array}$$

where *i* is not actually varying from 1 to *N*_*s*_. If the particle set has changed, the last particle weights of this set $w_{k-1,l}^{j}$ are not meaningful, and they must be reset as (1/*N*_*s*_). At last, the new temporal evidence set needs to be normalized again due to the following computation with the spatial evidence.

#### 3.2.2. Spatial Evidence

With the mean vector **μ*T*_*k,l*_** and the covariance matrix **Σ*T*_*k,l*_** of each temporal evidence matrix having been computed, the probabilities of all particle pixels $x_{k,l}^{j}$ can be calculated from the Gaussian distribution, and this probability are defined spatial evidence of each particle as

(6) $$\begin{array}{r} ES_{i,k,l}=N\left(x_{k,l}^{j}|\mu T_{k,l},\Sigma T_{k,l}\right) \end{array}$$

where *i* and $x_{k,l}^{j}$ should present the same pixel, and the none-particle pixels can be set to 0. Clearly, the larger spatial evidence this particle has, the more likely it belongs to the real LED markers. At last, the spatial evidence in each particle set are also needed to be normalized.

#### 3.2.3. Conditional Resampling

After updating the particle weights at the end of each window cycle, we should check the degeneration degree of each particle set which determine if a resampling is necessary, using the following equation where *Th*_*eff*_ is the threshold.

(7) $$\begin{array}{r} N_{eff}=\frac{1}{\overset{N_{s}}{\underset{j=1}{\sum }}{\left(w_{k,l}^{j}\right)}^{2}}<Th_{eff}\times N_{s} \end{array}$$

### 3.3. Tracking Results

**Figure 4:**
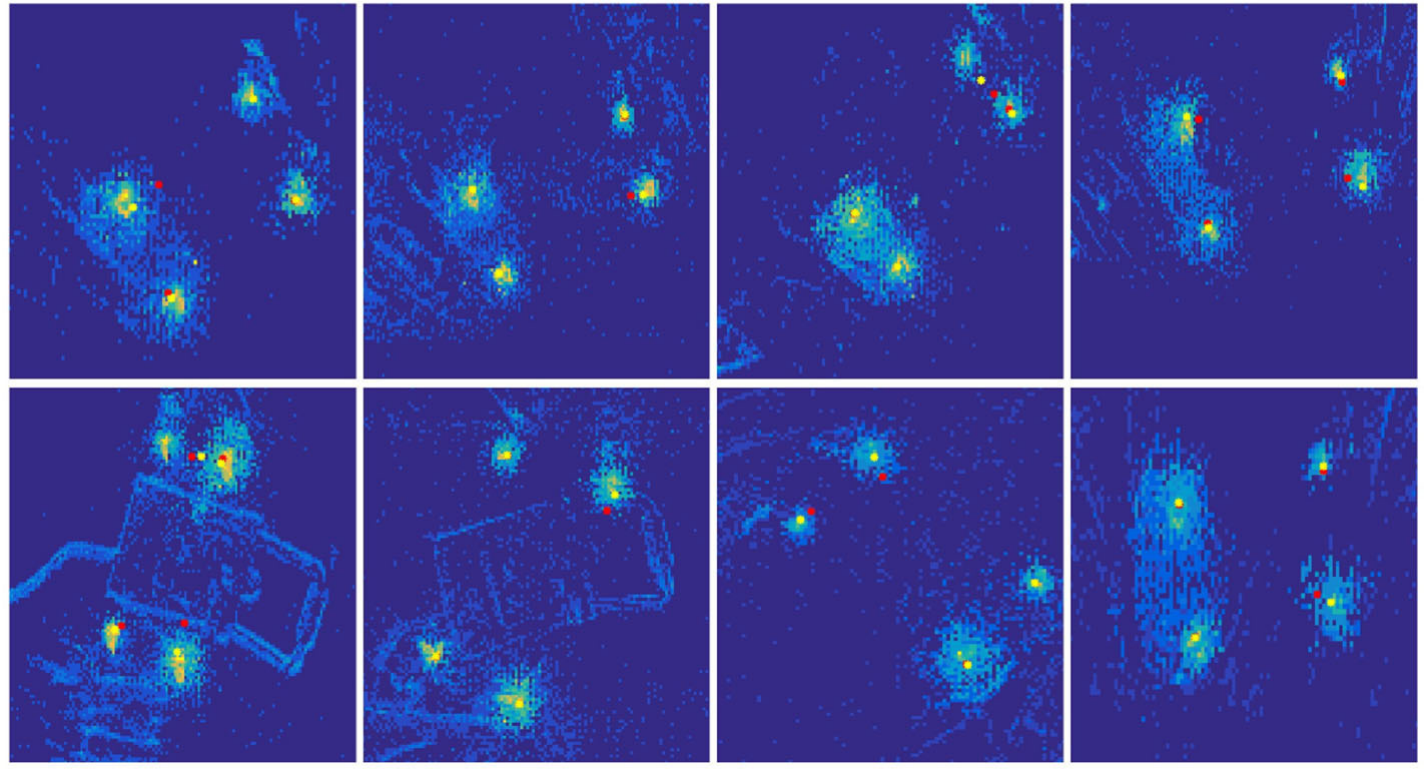
Comparing tracking results between spatiotemporal mixed particle filter and general particle filter. The yellow stars present the tracked LED positions from spatiotemporal mixed particle filter; the red stars present the tracked LED positions from general particle filter (only with temporal evidence).

**Figure 5:**
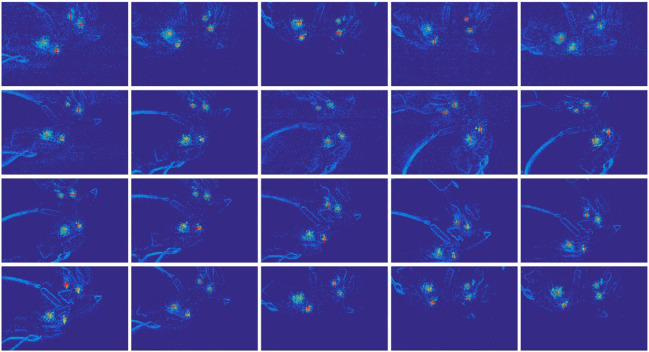
Tracking results of four LED markers along time. The markers are used to define the bad grasping position of a glue gun. Each subplot demonstrates the heat map of events recorded in the cycle window. The heat maps update in every cycle (1 ms), but they are only plotted every 250 ms for the demonstration here. The four red stars in each subplot represent the tracked marker positions.

The comparing results demonstrates in Figure 4. Obviously, the particle filter can track the LED markers with a better result. In an offline test, the reflection noise and the cross-impact noise will also destroy the tracking results due to the uncertainty of the object surface and multiple frequencies. These noises can be filtered by an SMP filter, and the results are shown Figure 5. We also used a general particle filter to track the LED markers in the same event data stream as the SMP filter. We compared the tracking results of both particle filter on 20 random object annotation data where each object has good grasping annotation and bad grasping annotation. Since the LEDs' position varies periodically, the tracked results are also supposed to change periodically. If the tracking results can fit the LEDs' event cluster in the image, the result is considered successful.

In the experiment, we consider tracking is failed when both markers are tracked together more than 100 cycles. The tracking results show that the general particle filter has 13 failed tracking trails in 40 trials, and our SMP filter only has 6 failed tracking trials in 40 trials. Besides, there are also 4 tracking trials using general particle filter during which one of the markers is never tracked on correct position along the whole stream; with our SMP filter, this situation never happened over the course of 40 trials. From Figure 5, it is clear that our tracking algorithm can perfectly track 4 LED markers at different sampling time points. Besides, the SMP can quickly and precisely track the 4 LED markers. The significant advantages of DAVIS contribute to a precise tracking result with a sub-ms-cycle level.

## 4. Event-Grasping Dataset

In this section, our method to construct the dataset used for training the model is presented. The whole training dataset consists of two parts:

- **Base dataset:** Base dataset is built using three event-to-frame encoding approaches;
- **Annotation dataset:** Annotation dataset is generated from LED markers tracking with SMP filter, which represent either good or bad grasping bounding box, and is then extended from single-grasp to multi-grasp.

After mapping these two dataset parts together cycle by cycle, the whole dynamic neuromorphic grasping dataset of high time resolution is constructed. Table 1 shows a summary of public dataset and our Event-Grasping dataset. Both Dex-net and Jacquard dataset are created by simulation, so that they can produce more synthetic data. Our dataset is similar to the Cornell dataset, which is recorded under real-world experiment environment. Compared with a manually annotated grasping dataset, our dynamic neuromorphic grasping system can annotate each grasping pose automatically with bounding box dynamically in millisecond.

**Table 1:** Summary of grasping datasets.

**Dataset**	**Modality**	**Objects**	**Images**
Cornell	RGB-D	240	885
Dexnet	Depth	1,500	6.7M
Jacquard	RGB-D	11K	54K
Event-grasping	Event stream	91	18.2K

### 4.1. Base Dataset

In our work, an event-to-frame conversion is carried out to construct the grasping base dataset consisting of around 91 objects. We are employing three encoding methods (Chen et al., 2019): *Frequency, SAE (Surface of Active Events), and LIF (Leaky Integrate-and-Fire)*, as introduced in this section, to process the continuous DAVIS event stream into a sequence of event frames for better use with deep learning algorithms. With time step of 20*ms*, a sliding window of specific interval over the whole event stream is used. Accumulative event information within the sliding window contributes to the generation of one frame as the window slides toward the end. We conducted our data collection in both high and low light conditions and encoded with a sliding window interval of 20*ms*.

#### 4.1.1. Event-Stream Encoding Based on Frequency

Given that more events would occur near an object's edges because edges of a moving object tend to be the edges of the illumination in the image, we utilized the event frequency as the pixel value to strengthen the profile of the object. At the same time, noise caused by the sensor could be significantly filtered out due to its low occurrence frequency at a particular pixel within a given time interval. Concretely, we counted the event occurrence at each pixel (*x, y*), and used this to calculate the pixel value using the following range normalization equation inspired by (Chen, 2017):

(8) $$\begin{array}{r} \sigma \left(n\right)=255\cdot 2\cdot \left(\frac{1}{1+e^{-n}}-0.5\right) \end{array}$$

where *n* is the total number of the occurred events (*positive* *or* *negative*) at pixel (*x, y*) within given interval, and σ(*n*) is the value of this pixel in the event frame, the range of which is normalized between 0 and 255 in order to fit 8-bit image.

#### 4.1.2. Event-Stream Encoding based on SAE (Surface of Active Events)

In order to take full advantage of the unique characteristic that neuromorphic vision sensors can record the exact occurring time of incoming events with low latency, the SAE (Surface of Active Events) (Mueggler et al., 2017) approach is applied to reflect time information while the pixel value and its gradient can tell the moving direction and speed of the event stream. Specifically, regardless of the event polarity, each incoming event [*t, x, y, p*] will change the pixel value *t*_*p*_ at (*x, y*) according to the time-stamp *t*. In this way, an event frame is acquired according to the time-stamp of the most recent event at each pixel:

(9) $$\begin{array}{r} SAE:t\Rightarrow t_{p}\left(x,y\right) \end{array}$$

Moreover, to attain an 8-bit single channel image, numerical mapping is conducted by calculating the Δ*t* between the pixel value *t*_*p*_ and the initial time *t*_0_ for each frame interval *T* as follows:

(10) $$\begin{array}{r} g\left(x,y\right)=255\cdot \frac{t_{p}-t_{0}}{T} \end{array}$$

#### 4.1.3. Event-Stream Encoding Based on LIF Neuron Model

According to the LIF (Leaky Integrate-and-Fire) neuron model (Burkitt, 2006), we regard every image pixel (*x, y*) as a neuron with its Membrane Potential (MP) and firing counter *n*. The MP value of a neuron will be influenced either by input spikes or time-lapse. In detail, each incoming event at pixel (*x, y*), regardless of polarity, will cause a step increase of this pixel's MP value. Simultaneously, the MP value of each pixel will decay at a fixed rate. When MP value of a pixel exceeds the preset threshold, a firing spike output will be generated there, and the MP value of this pixel will be reset to 0 with no latency. In a specific time interval, we count the number of times that a firing spike output is generated for each pixel (recorded as firing counter *n*). Then we carry out range normalization by using Equation (1) to acquire the corresponding pixel value. After each interval, the firing spikes counter *n* of each pixel is then reset to 0.

The reason why we employ these three encoding methods lies in their ability to reflect different characteristics of event data. In particular, the event frequency is used as pixel value, the edges of grasping object can be strengthened, which is benefit for grasping detection as we can get a clear profile of grasping object; SAE reflects time information of raw event; and the historical information of raw events can be represented by LIF model with time-continuous memory. The computational complexity of the three algorithms is efficient.

### 4.2. Annotation Dataset

#### 4.2.1. Grasping Rectangle

There are four edges in a parallelogram, including two grasping edges and two auxiliary edges. Grasping edges are used to place both parallel plates of the gripper. Four LED markers are numbered *l*_1_, *l*_2_, *l*_3_, and *l*_4_ and correspond to blinking frequencies. In our situation, the grasping edges are constructed by (*l*_1_, *l*_2_) and (*l*_3_, *l*_4_); the auxiliary edges are constructed by (*l*_1_, *l*_3_) and (*l*_2_, *l*_4_). We define a grasping parallelogram using coordinates of four parallelogram vertexes, which can be recalculated from some easily obtained information: the coordinates of two center points (*x, y*), the average slope, and the average length of both grasping edges.

**Figure 6:**
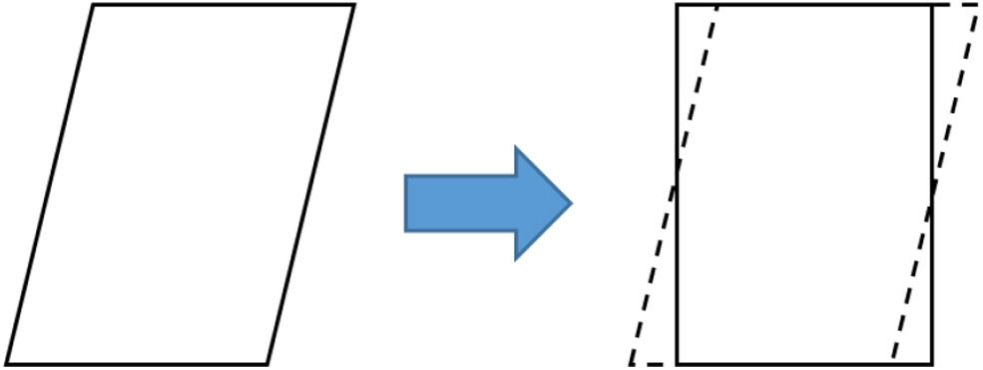
Parallelogram is transformed to rectangle. The whole area holds the same.

**Figure 7:**
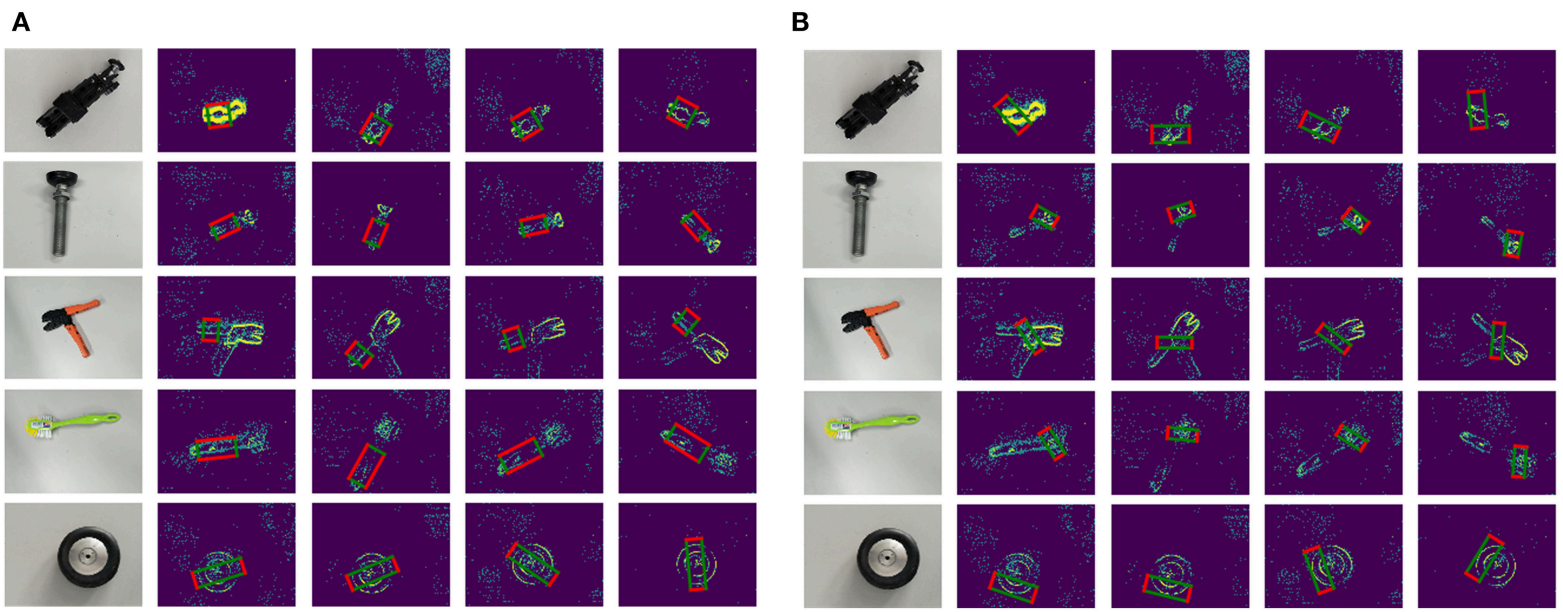
Single-grasping Annotations: **(A)** Good-grasping parallelogram annotations on original objects' event data; **(B)** Bad-grasping parallelogram annotations on original objects' event data. There are five typical objects selected, and there are four sampled time points in each recorded moving period. The first column presents the real grasping objects in traditional RGB image; the rest columns present the annotations and original objects' event data. The red edges present the grasping edge; and the green edges present the auxiliary edges.

Comparing the good grasping positions with the bad results, both annotations are plotted on the original object event data with the same sampled time points, shown as Figures 7A,B. In these results, there are five typical objects selected, and there are four sampled time points in each recorded moving period. The first column presents the real grasping objects in traditional RGB image; the rest columns present the annotations and original objects' event data. The red edges present the grasping edge, and the green edges present the auxiliary edges. Then we reshape a parallelogram to a rectangular by keeping the length of grasping edges same and translating both grasping edges along their straight lines until $\bar{\left(l_{1},l_{2}\right)}⊥\bar{\left(l_{1},l_{3}\right)}$, shown as Figure 6. A rectangular annotation consists of *x*, *y* coordinates of four LED markers: coordinates of four apexes on a rectangular.

#### 4.2.2. Multi-Grasping Annotation

After we represent the tracked LED points with rectangles, each event frame acquires the ground truth of a good grasping position and a bad grasping position, as is shown in Figure 7. However, the ground truth grasps of an object tend to be multifarious, and this is also an indispensable requirement for successful training of grasp detection. The large workload of manual grasp annotation is a perennial problem, let alone the huge data amount of low-latency event streams. In this context, we proposed an approach to automatically generate multi-grasping annotation using the previous tracking results.

Since the eight tracked LED points (regardless good or bad positions) are in the same plane, they conform to the coplanar constraint; that is to say, the homography matrix between any event frame and the first frame can be calculated. As is explained in detail in Hartley and Zisserman (2003), the homography matrix directly represents the transformation relationship between two coplanar image coordinates. We have a camera looking at points *P*_*i*_ at two different positions *A* and *B*, which generate the projection points $p_{i}^{A}=\left(u_{i}^{A},v_{i}^{A},1\right)$ in *A* and $p_{i}^{B}=\left(u_{i}^{B},v_{i}^{B},1\right)$ in *B*. The transformation relationship between the projections is:

(11) $$\begin{array}{r} p_{i}^{B}=K\cdot H_{BA}\cdot K^{-1}\cdot p_{i}^{A} \end{array}$$

**Figure 8:**
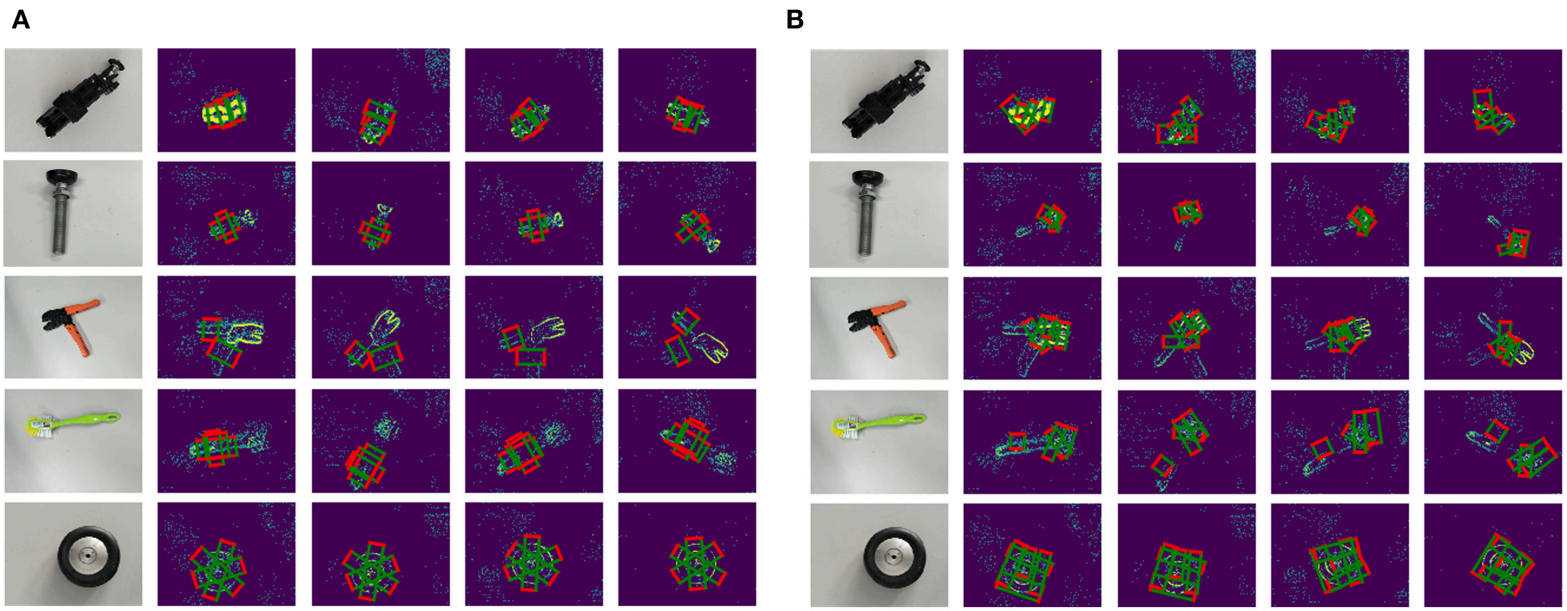
Multi-grasping Annotations: **(A)** Good grasping parallelogram annotations on original objects' event data; **(B)** Bad grasping parallelogram annotations on original objects' event data. There are five typical objects selected, and there are four sampled time points in each recorded moving period. The first column presents the real grasping objects in traditional RGB image; the rest of the columns present the annotations and original objects' event data. The red edges present the grasping edge, and the green edges present the auxiliary edges.

With the Equation (11), the homography matrix between two event frames can be calculated at least with four matched points. Therefore, we utilize the eight matched LED points to calculate the coefficient matrix $K\cdot H_{BA}\cdot K^{-1}$ and applied RANSAC algorithm to reduce errors. Afterwards, as long as we manually set ground truth annotations in the first frame, the transformed annotations in any other frame can be attained. Besides, we also applied the spatial particle filter similar with section 3 in order to smooth the trajectories and enhance accuracy. The final dataset with multi-grasping annotations are illustrated in Figure 8.

## 5. Grasping Detection Method

**Figure 9:**
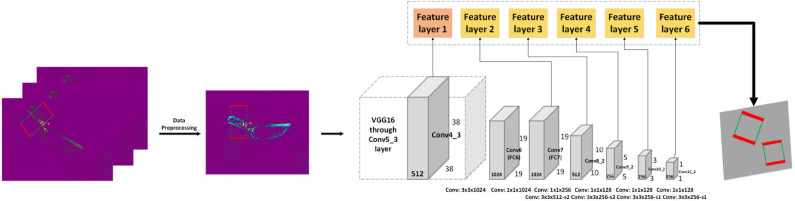
The structure of our grasping detection system. The event streams are processed by three encoding methods and merged into a three-channel event frame, which is then fed into the grasping algorithm. The backbone network extracts the features, and six feature maps are employed for grasp pose estimation. Gray blocks indicate network layers and color blocks represent feature layers.

In this section, a model is introduced to detect grasping bounding box proposals on given event frames of single object. The overall architecture of the system is shown in Figure 9. The event frames are processed and taken as input for the network. Much like many state-of-art object-detection algorithms, our grasping detection system adopts VGG16 to extract feature. Then, the network makes classification and bounding box regression on multiple feature maps and combines predictions from feature maps to handle grasping object detection with different resolutions.

### 5.1. Data Pre-processing

In this paper, we consider orientation of grasping rectangle as angle classification problem. After deploying data pre-processing methods on our event frame and annotation data, algorithms in object detection can be transferred to grasp detection, as shown in Figure 9.

#### 5.1.1. Event Frame Pre-processing

To take full advantage of the event information from neuromorphic vision sensor, we merged three corresponding event frames using three different event-stream encoding methods, Frequency, SAE and LIF mentioned in section 4.1. The definition of merged frame is [*R, G, B*] = [*Frequency, SAE, LIF*]. We normalized event information encoded by Frequency, SAE, and LIF to the range of 0 to 255 and substituted the red channel with the Frequency, the green channel with the SAE, and the blue channel with the LIF, respectively.

#### 5.1.2. Ground Truth Pre-processing

In our Event-Grasping dataset, the annotation of the bounding box is a rectangle with an inclined angle. To normalize the input of proposed grasping network, the orientated ground truth bounding box is reset to have vertical height and horizontal width before feeding in neural network, as shown in Figure 9.

### 5.2. Proposals Detection With Multi-Scale Feature Map

The architecture of Liu W. et al. (2016) introduced in the area of object detection is applied for our grasping network, and VGG16 (Simonyan and Zisserman, 2015) pre-trained on ImageNet is used to extract features. The input image size of our network is 300 × 300, and the size of feature maps is *N* × *N* with *k* channels. At each of the *N* × *N* locations, a 3 × 3 × *k* small convolutional kernel is applied to produce scores for grasping angle category or the shape offset of a grasping rectangle bounding box. Based on feed-froward convolutional network, multi-Scale feature map also is used for grasping detection. In Figure 9, six different feature layers are applied to predict grasping object at multiple scales. Each feature layer can do angle classification and bounding box regression. Besides, the feature map cell tiles a set of default bounding boxes with convolutional manner. In each feature map cell, different shape default boxes are used for grasp reset bounding box shape variations. By combing default bounding box and multi-scale feature map, the network can achieve better detection accuracy and detect small grasping object efficiently.

### 5.3. Classification for Grasping Orientation

In many previous works (Redmon and Angelova, 2015; Kumra and Kanan, 2016), the authors have regressed a single 5-dimensional grasp representation {*x, y, w, h*, θ} for grasping detection. The drawback of angle regression is singularities which occur in single angle learning process. In this work, instead of regression used in prior approaches, we defined grasping detection to be angle classification and grasp rectangle bounding box regression. We divided the grasp representation orientation coordinate θ into *C* equal sizes and transform grasp orientation problem into classification task. Specifically, we equally quantized 180 degrees into *C* intervals and each interval associated grasp orientation is assigned to a class label. Additionally, since collecting orientation class may be a non-grasp orientation, we added a label *l* = 0 to represent it. The label *l*_*i*_ is the associated corresponding grasp orientation angle θ_*i*_, and *l*_*i*_ ∈ 1,.,C. The total number of labels is |*L*| = *C* + 1. As in Chu et al. (2018), *C* = 19 is utilized in this paper.

### 5.4. Loss Function

Loss function of our grasping network includes two parts: grasping orientation angle classification loss *L*_*c*_ and grasping rectangle bounding box regression loss *L*_*r*_. The grasping rectangle regression loss is a Smooth L1 loss. Our network regress four dimensions offset of each default bounding box as shown in Equation (12).

(12) $$\begin{array}{r} L_{r}\left(x,p,g\right)=\overset{N}{\underset{i\in pos}{\sum }}\underset{m\in \left\{cx,cy,w,h\right\}}{\sum }x_{ij}^{k}smooth_{L1}\left(p_{i}^{m}-g_{j}^{m}\right) \end{array}$$

where *N* is the number of positive oriented default bounding boxes. (*c*_*x*_, *c*_*y*_) is the center of the default bounding box, and its width and height represent *w* and *h*, respectively. $p_{i}^{m}$ is the offset predicted by the network. $g_{j}^{m}$ is the corresponding ground truth offset value. The grasping orientation angle classification loss is the softmax loss, as shown in Equation (14).

(13) $$\begin{array}{r} L_{c}\left(x,c\right)=-\overset{N}{\underset{i\in pos}{\sum }}x_{ij}^{p}\log{ĉ}_{i}^{n}-\underset{i\in neg}{\sum }\log{ĉ}_{i}^{0} \end{array}$$

(14) $$\begin{array}{c} where\;{\hat{c}}_{i}^{n}=\frac{\exp(c_{i}^{n})}{\sum_{n}\exp(c_{i}^{n})} \end{array}$$

Finally, the total loss for grasping detection is

(15) $$\begin{array}{r} L\left(x,c,p,g\right)=\frac{1}{N}\left(L_{c}\left(x,c\right)+\alpha L_{r}\left(x,p,g\right)\right) \end{array}$$

## 6. Experiments and Results

We evaluated our grasping detection algorithm on the Event-Grasping dataset, which is recorded with neuromorphic vision sensor (DAVIS) in two different light conditions, light and dark. We will discuss the impact of different brightness on grasping detection accuracy. Like previous works, the convolutional layers for feature extraction are pre-trained on ImageNet.

### 6.1. Training

In training period, we train the grasping network end to end for 240 epochs on two Nvidia GTX1080Ti GPUs with 22GB memory. The batch size is set as 32. SGD is used for optimizing our model. Additionally, we define the initial learning rate as 0.002. MXNET is the implementation framework with cudnn-5.1.10 and cuda-8.0 packages.

### 6.2. Metrics

Like Kumra and Kanan (2016) and Chu et al. (2018), rectangle metric is used in this paper to evaluate grasping detection results. A prediction of grasp configuration is regarded as correct if it satisfies both:

- **Angle difference:** The difference of rotated angle between the predicted grasp and ground truth is within 30°.
- **Jaccard index:** The Jaccard index of ground truth and the predicted grasp is >25%, as shown in Equation (16).
  (16) $$\begin{array}{r} J\left(g_{p},g_{t}\right)=\frac{\left|g_{p}\cap g_{t}\right|}{g_{p}\cup g_{t}} \end{array}$$
  where *g*_*p*_ represents the area of the predicted grasp rectangle, and *g*_*t*_ represents the area of the ground truth. *g*_*p*_ ∩ *g*_*t*_ is the intersection of predicted grasp rectangle and ground truth rectangle. *g*_*p*_ ∪ *g*_*t*_ is the union of predicted grasp rectangle and ground truth rectangle.

Furthermore, the dataset is divided into two levels for evaluating the generalization ability of the model:

- **Image-wise level:** The dataset is randomly divided into training set and test set. The training set and test set do not share the same image of each grasp object.
- **Object-wise level:** All the images of one object are divided into the same set (training set or test set), which is to validate the generalization ability of our grasping network for unseen object.

### 6.3. Results

The proposed architecture was validated on our Event-Grasping dataset. We explored the performance of neuromorphic vision sensor (DAVIS) in different light conditions and analyzed the experiment results of our grasping detection algorithm at different evaluate metric thresholds. We chose the highest score generated from the proposed detection grasping approach as the final output. Afterwards, we used the rectangle metric mentioned above in section 6.2 to evaluate our grasping detection system. The grasping detection results are shown in Tables 2, 3.

**Table 2:** Detection accuracy (%) at different Jaccard thresholds (angle threshold is 30°).

**Light condition**	**Jaccard thresholds**							
	**Image-wise**				**Object-wise**			
	**25%**	**30%**	**35%**	**40%**	**25%**	**30%**	**35%**	**40%**
Light	97.8	97.3	96.7	96.2	93	92.5	91	85
Dark	96.2	96.2	95.1	95.1	92.5	91.5	89	84.5

**Table 3:** Detection accuracy (%) at different angle thresholds (Jaccard threshold is 25%).

**Light condition**	**Angle thresholds**							
	**Image-wise**				**Object-wise**			
	**15°**	**20°**	**25°**	**30°**	**15°**	**20°**	**25°**	**30°**
Light	96.7	97.8	97.8	97.8	84.5	90	90	93
Dark	92.8	95.6	95.6	96.2	81.5	88	88	92.5

Table 2 contains the outputs of the model with different Jaccard thresholds, and Table 3 includes the outputs of the model with different Angle thresholds. The performance of the proposed grasping detection method on image-wise split presents the network can estimate grasp pose of the new position of objects which it has been seen before. For object-wise split, this dataset split approach tests the ability of the grasping network to generalize to the unseen grasp objects. With the stricter evaluate metrics, the model still achieves better detection precision and reach 93% accuracy at an objects-wise split. The experiment results indicate that the generalization ability of the model for unseen object is performed well. The inference time of our algorithm on 1080TI is 20 ms, and the saved weight parameter is 100 MB, which has a good real-time performance. Moreover, by comparing the different light conditions, we can see that neuromorphic vision sensor is sensitive to light intensity and performs well under high illumination intensity.

**Figure 10:**
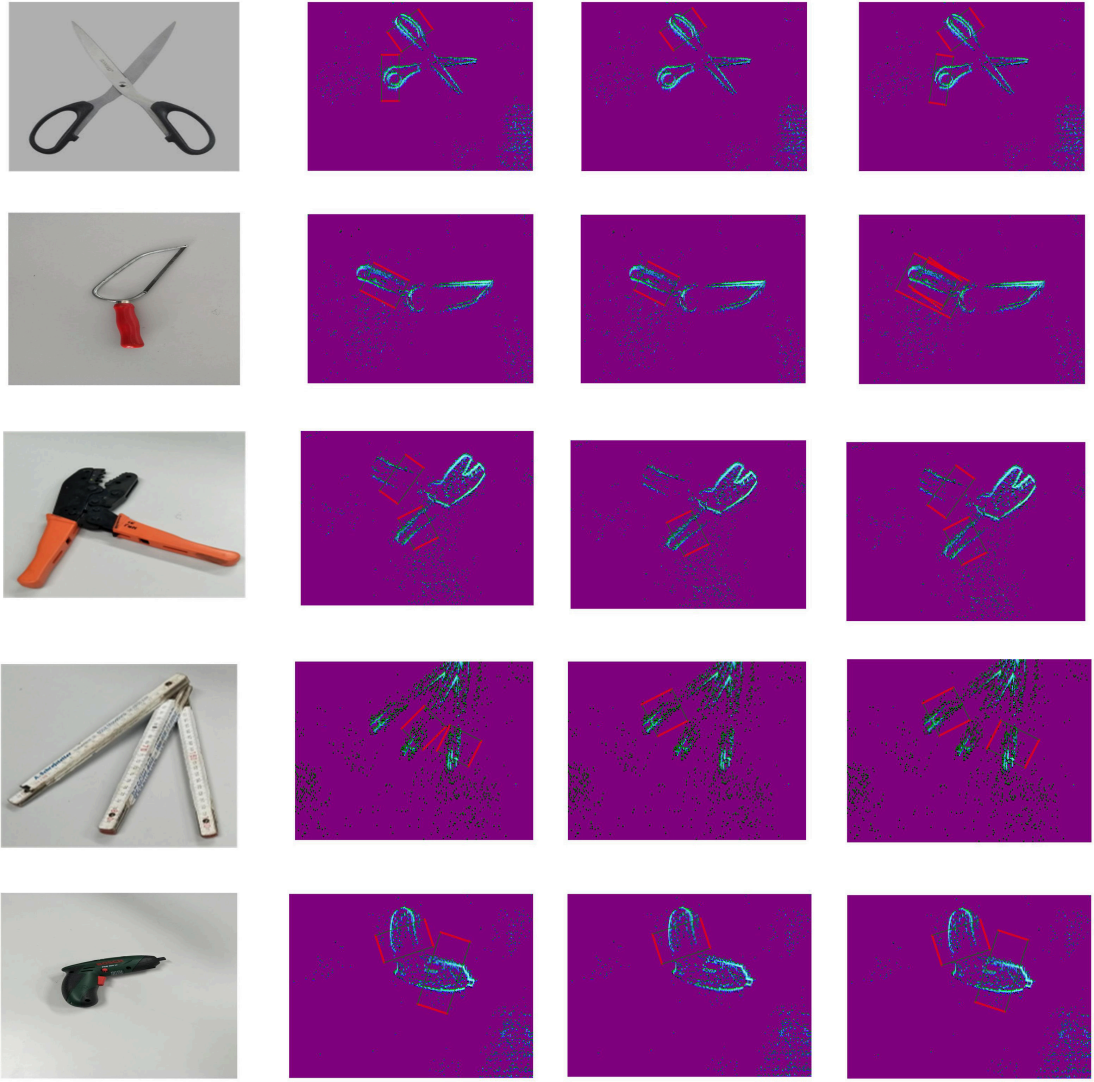
The detection results from grasping network. The first column is the RGB images of grasping objects. The second column is the ground truth of grasping rectangle. The third column is the top grasp outputs for several objects. The last column is the multi-grasp results.

In Figure 10, the grasping detection results of some objects are plotted. The first column presents the real grasping object in RGB image. The ground truth grasping rectangle of the objects are presented in the second column. By limiting the output to a single grasp, the top-1 detection results are visualized in the third column. Furthermore, the multi-grasp results are depicted in the fourth column. In the multi-grasp case, our grasping detection model predicts grasping rectangle from the feature of different objects rather than just learned from ground truth. The detection results of these objects demonstrate that our grasping detection system can predict grasp configuration efficiently and have a better generalization ability.

### 6.4. Discussions

**Figure 11:**
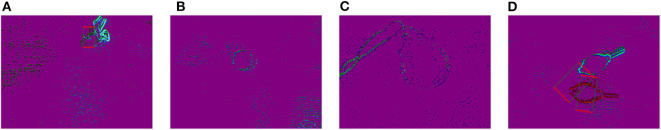
Failed detection cases: **(A)** detection of failure; **(B,C)** no detection results at a threshold; and **(D)** an anomaly in the event sensor causes a detection error.

To the best of our knowledge, this work is a first application using neuromorphic vision sensor for robotic grasping under a real-world environment, which makes it suited as a benchmark for further event-based researches. In this work, we only studied event-based grasping detection, and there are still many potential research directions, such as improving grasping detection accuracy by fusing event data and frame images. Some failed detection cases are presented in Figure 11. The edge of some objects with dense events will lead to false detection, and the algorithm proposed in this work is not very good for the recognition of some grasping objects with circular contours, which may be caused by the fact that the circular grasping objects are difficult to generate sufficient events to reflect their shape contour information. In addition, as Figure 11D shows, an occasional anomaly from the event sensor can cause detection to fail.

## 7. Conclusion

In this paper, we constructed a dynamic robotic grasping dataset using neuromorphic vision sensor (DAVIS), which contains around 91 generic objects. Our dataset construction method largely reduces the time and labor resources and provide dynamic annotation results at the time resolution of 1 ms. Based on this dataset, we also introduced a single deep neural network for grasping detection with combing predictions from multiple feature maps. Our grasping detection algorithm can achieve a high detection accuracy with 93% precision at object-wise level.

## Data Availability Statement

The datasets presented in this study can be found in online repositories: https://github.com/HuCaoFighting/DVS-GraspingDataSet.

## Author Contributions

BL and HC did the conception and design of the manuscript. BL, HC, ZQ, YH, and ZW did the analysis and interpretation of data, drafting, and revising the article. HC, ZL, ZW, and ZQ acquired the data. All authors contributed to the article and approved the submitted version.

## Conflict of Interest

BL is employed by the company JingDong Group. The remaining authors declare that the research was conducted in the absence of any commercial or financial relationships that could be construed as a potential conflict of interest.
